# Predictors of Mortality among United States Veterans with Human Immunodeficiency Virus and Hepatitis C Virus Coinfection

**DOI:** 10.1155/2014/764540

**Published:** 2014-04-07

**Authors:** Sebhat Erqou, Arpan Mohanty, Pashtoon Murtaza Kasi, Adeel A. Butt

**Affiliations:** ^1^Department of Medicine, Division of Hospital Medicine, Weill Cornell Medical College, 525 East 68th Street, P.O. Box 130, New York, NY 10065, USA; ^2^VA Pittsburgh Health Care System, Pittsburgh, PA 15213, USA; ^3^Department of Medicine, Yale School of Medicine, New Haven, CT 06510, USA; ^4^Department of Medicine, Mayo Clinic, Rochester, MN 55905, USA; ^5^Department of Medicine, Sheikh Khalifa Medical City, Abu Dhabi, UAE; ^6^Department of Medicine, University of Pittsburgh School of Medicine, Pittsburgh, PA 15213, USA

## Abstract

*Background*. Understanding the predictors of mortality in individuals with human immunodeficiency virus and hepatitis C virus (HIV/HCV) coinfection can be useful in management of these patients. * Methods*. We used the Electronically Retrieved Cohort of HCV Infected Veterans (ERCHIVES) for these analyses. Multivariate Cox-regression models were used to determine predictors of mortality. * Results*. Among 8,039 HIV infected veterans, 5251 (65.3%) had HCV coinfection. The all-cause mortality rate was 74.1 (70.4–77.9) per 1000 person-years (PY) among veterans with HIV/HCV coinfection and 39.8 (36.3–43.6) per 1000 PY for veterans with HIV monoinfection. The multivariable adjusted hazard ratio (95% confidence interval) of all-cause mortality for HCV infection was 1.58 (1.36–1.84). Positive predictors of mortality included decompensated liver disease (2.33 (1.98–2.74)), coronary artery disease (1.74 (1.32–2.28)), chronic kidney disease (1.62 (1.36–1.92)), and anemia (1.58 (1.31–1.89)). Factors associated with reduced mortality included HCV treatment (0.41 (0.27–0.63)) and higher CD4 count (0.90 (0.87–0.93) per 100 cells/**μ**L higher count). Data were insufficient to make informative analyses of the role of HCV virologic response. * Conclusion*. HCV coinfection was associated with substantial increased risk of mortality among HIV infected veterans. HCV treatment was associated with significantly lower risk of mortality.

## 1. Background

Human immunodeficiency virus (HIV) is an important cause of morbidity and mortality globally. Worldwide, there are approximately 34 million people with HIV infection, 68% of which are in sub-Saharan African countries [1]. In 2010, there were 1.8 million deaths due to HIV/AIDS globally [1]. In the United States, there were about 1.1 million people infected with HIV in 2010 [2], and 16 thousand individuals died of HIV [2]. Approximately 30–40% of HIV infected persons are coinfected with hepatitis C virus (HCV) [3, 4]. This proportion is as high as 70% among individuals who use intravenous drugs [5].

Prior to the advent of highly active antiretroviral therapy (HAART) the outcome of HIV infected individuals has been largely the function of progression to acquired immunodeficiency syndrome (AIDS) and development of opportunistic infections. In the HAART era, the longevity of HIV infected individuals has significantly improved and chronic conditions such as HCV infection are emerging as important factors in morbidity and mortality [6–8].

HIV/HCV coinfected individuals develop the complications of HCV infection (i.e., cirrhosis of liver, end-stage liver disease, and hepatocellular carcinoma) more rapidly than those with HCV monoinfection [9, 10]. Similarly, some studies have shown that the incidence of AIDS defining illnesses are increased among HIV/HCV infected individuals compared to those with HIV monoinfection [11–13], while others have not confirmed this [7, 13]. Regardless of the specific outcomes, it is well established that individuals with HIV/HCV coinfection have significantly higher morbidity and mortality compared to individuals with HIV monoinfection [7, 12, 13]. However, specific predictors of mortality and how they differ in the HIV/HCV coinfected versus HIV monoinfected individuals in the era of HAART have not been well documented.

Understanding the predictors of mortality in HIV/HCV coinfected individuals can help identify potential targets for interventions that may improve survival. We analyzed data from the Electronically Retrieved Cohort of HCV Infected Veterans (ERCHIVES) study to determine the incidence of all-cause mortality and its predictors among HIV/HCV coinfected individuals and HIV monoinfected counterparts.

## 2. Patients and Methods

ERCHIVES is a retrospective cohort of HCV infected individuals and HCV uninfected controls. Version 2 of ERCHIVES, which was used for the current analyses, was assembled and merged from 2001 to 2008 through linkage of records from several sources of the Veterans Health Administration Healthcare System (VA) [14–18]. Demographic and clinical data were retrieved from the Department of Veterans Affairs National Patient Care Database, pharmacy data from the Pharmacy Benefits Management Database, laboratory data from the Decision Support System and the Corporate Data Warehouse, and mortality data from the Beneficiary Identification Records Locator Subsystem. HCV infected individuals were identified by a positive HCV antibody at baseline. Corresponding HCV uninfected controls were identified by a negative HCV antibody test performed in the same year as a positive antibody test for the cases and were matched by age (5-year blocks), race, sex, and geographic location. HIV infected individuals were identified by positive HIV antibody test during routine clinical care. For the current study, we included subjects with HIV monoinfection and HIV/HCV coinfection.

Baseline demographics and comorbidities, including smoking, alcohol and drug abuse or dependence, hypertension, diabetes, chronic kidney disease (CKD), decompensated liver disease, chronic obstructive pulmonary disease (COPD), cancer, coronary artery disease (CAD), peripheral vascular disease (PVD), and stroke, were defined as described previously [15–20]. Comorbidities were considered as baseline if first recorded prior to, or within 6 months after, entry to cohort. Patients with decompensated liver disease at baseline were identified using a combination of ICD codes and laboratory parameters including elevated INR (>1.7) or bilirubin (>2 g/dL) or lower albumin (<2.5 g/dL) concentrations, as previously described [21]. Laboratory data retrieved included hemoglobin, white blood cell count, total cholesterol, low-density and high-density lipoprotein cholesterol, triglycerides, alanine aminotransferase (ALT), aspartate aminotransferase (AST), and HCV and HIV antibody tests. A subset of participants had available information on CD4 counts, HIV RNA levels and/or HCV RNA levels. Anemia was defined as hemoglobin level less than 13 g/dL or 12 g/dL for men and women, respectively. Due to limitations in data, virologic response was defined as undetectable HCV RNA level on treatment, at the end of treatment, or after treatment, for the purpose of this study.

Data on use of interferon alpha, pegylated interferon alpha, and ribavirin in various combinations was obtained from the Pharmacy Benefits Management Database. Dates of starting treatment and cumulative durations of prescription were obtained. Patients with interferon doses higher than those routinely used for HCV treatment were excluded [22]. All-cause mortality data were obtained from VA Beneficiary Identification Records Locator System. Subjects were followed up until death or the last observation date in the cohort. Individuals with no follow-up visits after baseline visit were excluded from analyses.

Baseline characteristics of HIV/HCV coinfected individuals were compared to those with HIV monoinfection using *t*-test for continuous variables and chi-square test for categorical variables. Survival analyses were used to determine the associations of HCV coinfection status and mortality. The Kaplan-Meier plots were used to visually compare survivor function by HCV status. Log-rank test was used to determine the statistical significance of differences between survivor curves. The hazard ratios (HRs) and 95% confidence intervals (CIs) for the association of HCV status with mortality among HIV infected individuals were estimated using the Cox proportional hazard models with adjustment for confounders. Predictors of mortality were investigated among individuals with HIV/HCV coinfection and those with HIV monoinfection using the Cox models. Covariates included in the multivariate Cox model were (i) sociodemographic factors including age, sex, race, smoking, alcohol, and drug abuse, (ii) baseline comorbidities, including diabetes, hypertension, decompensated liver disease, CKD, COPD, cancer, CAD, stroke, and PVD, and (iii) CD4 count, HIV RNA level, and HCV treatment status. The assumptions of the proportionality of hazards were evaluated using the Schoenfeld residuals. All analyses were performed using Stata (College Station, TX), version 9.

## 3. Results

The baseline characteristics of the participants are shown in Table 1. Among 8,039 HIV infected veterans, 5251 (65.3%) had HCV coinfection. The mean (SD) age of participants was 50.0 (7.3) years; 98.2% were male, 29.5% White, 57.9% Black, and 7.4% Hispanic. Participants with HIV/HCV coinfection, compared to those with HCV monoinfection, were more likely to be Black (60% versus 54%) or smokers (20% versus 17%), have hypertension (32% versus 29%), diabetes (14% versus 11%), anemia (52 versus 44%), or decompensated liver disease (26% versus 20%), or have a history of drug abuse or dependence (39% versus 18%) or alcohol abuse or dependence (28% versus 16%) (*P* value < 0.01, for all comparisons). They were also more likely to have lower body mass index (24.7 versus 25.4 Kg/m^2^), lower levels of albumin (3.6 versus 3.87 g/dL), or higher levels of AST (70 versus 47 IU/L) or ALT (64 versus 46 IU/L) (*P* value < 0.001, for all comparisons). Of note, the groups were similar with regard to CD4 count (355 versus 366 cells/*μ*L, *P* value = 0.15) and HIV viral load (69800 versus 70100 copies/*μ*L, *P* value = 0.94).

**Figure 1:**
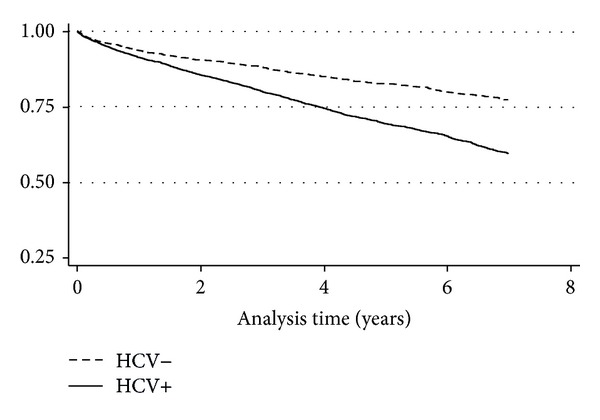
The Kaplan-Meier curve for HIV infected veterans by HCV infection status.

The all-cause mortality rate was higher among veterans with HIV/HCV coinfection (HR (95% CI), 74.1 (70.4–77.9) per 1000 person-years (PY)), compared to those with HIV monoinfection (39.8 (36.3–43.6) per 1000 PY) (Figure 1). The HR of all-cause mortality for HCV infection was 1.78 (95% CI, 1.59–1.96) in the age- and sex-adjusted Cox model. The association of HCV with mortality was attenuated by 7% after adjusting for baseline decompensated liver disease (1.66 (1.44–1.93)). The corresponding HR after adjusting for several baseline sociodemographic and comorbidity variables was 1.58 (1.36–1.84) (Table 2). The association was further reduced to 1.50 (1.28–1.75) after adjustment for incident cirrhosis. The association of HCV with mortality was 1.47 (1.21–1.79) after adjustment for baseline HIV RNA level in a subset with available data (Supplementary Table  1 in Supplementary Material available online at http://dx.doi.org/10.1155/2014/764540). Other baseline variables associated with mortality in the multivariable Cox model among veterans with HIV infection (with or without HCV coinfection) included decompensated liver disease (2.17 (1.88–2.50)), CAD (1.78 (1.42–2.23)), CKD (1.74 (1.50–2.02)), anemia (1.59 (1.36–1.86)), and cancer (1.50 (1.26–1.69)). By contrast, higher CD4 count (0.90 (0.88–0.93) per 100 cells/*μ*L higher count) and body mass index (0.94 (0.92–0.95) per 1 kg/m^2^) were associated with reduced mortality (Table 2). For comparison, the univariate association of these factors is shown in Supplementary Table  2.

Among veterans with HIV/HCV coinfection, decompensated liver disease (HR (95% CI) 2.33 (1.98–2.74)), CAD (1.74 (1.32–2.28)), CKD (1.62 (1.36–1.92)), anemia (1.58 (1.31–1.89)), COPD (1.57 (1.26–1.95)), cancer (1.52 (1.23–1.87)), and age (1.34 (1.21–1.49) for every 10-year-older age) were predictors of higher risk of mortality, while HCV treatment (0.41 (0.27–0.63)), CD4 count (0.90 (0.87–0.93) per 100 cells/*μ*L higher count), and Black race (0.72 (0.60–0.87)) were associated with significantly lower risk of mortality (Table 3). The associations were broadly similar among individuals with HIV monoinfection, except for stroke and diabetes which were strongly associated with mortality, while Hispanic race, thromboembolism, PVD, and drug abuse were inversely associated with mortality, in this group. Unlike in HIV/HCV coinfected veterans, COPD was not associated with mortality in those with monoinfection. For comparison, the univariate association of these factors by HCV status is shown in Supplementary Table  3. We explored the role of HCV virologic response in a subset of participants that had available data on HCV viral load (Table 4). Of the 469 people that had received HCV treatment, 137 (29%) had available data on HCV viral load, of which 21 (15%) had virologic response. Mortality rate was significantly reduced among individuals who received HCV treatment even when they did not achieve virologic response. The number of participants with virologic response was too small to make informative analysis of the association in this category (Table 4).

## 4. Discussion

In our present analyses involving over 8,000 HIV infected veterans, including 65% with HCV coinfection, we found a 75% increased risk of mortality among HIV/HCV coinfected veterans compared to those with HIV monoinfection. Decompensated liver disease, CAD, CKD, and anemia were associated with increased risk of mortality among individuals with HIV/HCV coinfection, while higher CD4 count and HCV treatment were associated with lower risk of mortality in this group.

In the pre-HAART era, the high rate of AIDS related mortality had masked the morbidity and mortality associated with HCV in HIV infected individuals. With the advent of HAART and improvement in the longevity of HIV infected individuals, HCV infection has emerged as an important cause of morbidity and mortality [4, 7, 12, 13, 23]. Several studies assessing individuals with HIV infection in the post-HAART era found a 1.4- to 2.5-fold increased risk of mortality compared with individuals with HIV monoinfection [7, 11, 12]. This is because HIV infection accelerates the course of HCV infection resulting in more rapid progression to cirrhosis and end-stage liver disease [4, 12, 24]. In addition, it has been proposed that the increased risk of cardiovascular and chronic kidney diseases in HCV may be mediated by the extrahepatic manifestations of hepatitis such as glomerulonephritis, insulin resistance, and cryoglobulinemia [23, 25]. On the other hand, whether HCV infection accelerates the course of HIV disease is unclear, with studies reporting conflicting findings [13, 26].

Consistent with previous reports we found substantially increased risk of mortality among HIV/HCV coinfected veterans compared to those with HIV monoinfection. HIV/HCV coinfected veterans had significantly higher prevalence of baseline comorbidities including decompensated liver disease, CKD, and anemia. The association was considerably attenuated with adjustment for several variables, in particular for decompensated liver disease. However, there was still a 60% highly statistically significant increased risk remaining in the fully adjusted model including 21 variables, indicating that confounding is not likely to explain the observed association. Further adjustment for incident cirrhosis reduced the association only by 10%, which suggests that the increased mortality in HIV/HCV coinfection may not be only mediated by increased progression to end-stage liver disease. We, however, did not have data on cause-specific mortality and hence were not able to explore this hypothesis further.

Understanding the predictors of mortality in individuals with HIV/HCV coinfection can be useful for targeting interventions that may help improve outcome. In the present study, we found several baseline comorbidities to be important predictors of mortality in HIV/HCV coinfected veterans. Decompensated liver disease, CKD, CAD, anemia, COPD, and cancer were each associated with 50–70% higher risk of death. On the other hand, higher CD4 count and body mass index and HCV treatment were associated with lower risk of mortality in this group. These findings highlight the role of antiretroviral therapy, HCV treatment, and improved nutrition in improving outcomes in HIV/HCV coinfected individuals. In particular, the 60% risk reduction noted for HCV treatment reinforces its importance in improving the survival of these patients [13]. The importance of this finding is heightened by the fact that about a third of the HIV infected patients are also coinfected with HCV [3, 4]. In our study, HCV treatment was associated with reduced mortality in those without virologic response and those without sufficient data to enable determination of the status of virologic response. The later observation may be because these individuals had achieved sustained virologic response or it could be that HCV treatment, regardless of virologic response status, improves survival. For instance, we have previously reported that even incomplete HCV treatment leads to survival advantage [14].

The strengths of the current report include the large size, involving 8000 HIV infected individuals of whom 5000 had HCV coinfection. Second, availability of a wide range of covariates including several baseline comorbidities enabled good control for confounding in assessing the association of HCV with mortality in this population. Third, the availability of information on several baseline comorbidity variables also allowed extensive investigation of predictors of mortality in HIV/HCV coinfected individuals. Fourth, use of mortality as an endpoint reduced the likelihood of any random or systematic misclassification.

Study limitations include analysis of administrative databases and the fact that data were not collected for the specific purpose of this study. Second, as we used all-cause mortality (as compared to liver related mortality), we were not able to determine the proportion of deaths directly attributable to HCV infection. Third, the predominantly male population of veterans with high burden of comorbidities may be somewhat different from other HIV/HCV coinfected populations, which might limit extrapolation to other settings. Fourth, we did not have sufficient HCV RNA data to make informative analysis of the role of virologic response in predicting mortality among individuals that received HCV treatment. Nonetheless, using data on a large number of HIV/HCV coinfected individuals for whom several baseline and follow-up variables were available, our study provides evidence of the importance of HCV coinfection in HIV infected patients and the role of HCV treatment and appropriate management of medical comorbidities in this population.

In conclusion, we found that HCV coinfection was associated with substantial increased risk of mortality in HIV infected veterans. The association persisted after taking into account several baseline comorbidity variables and incident cirrhosis. Decompensated liver disease, CAD, CKD, and anemia were associated with materially increased risk of mortality among individuals with HIV/HCV coinfection, while higher CD4 count and HCV treatment were associated with lower risk of mortality in this group. Our findings highlight the need for close monitoring of HIV/HCV coinfected patients with focus on providing them with HCV treatment and appropriate management of comorbidities, in addition to antiretroviral therapy.

## Supplementary Material

Supplementary Tables: these materials provide supplementary information to the manuscript “Predictors of mortality among United States veterans with human immunodeficiency virus and hepatitis c virus coinfection”.
Supplementary Table 1: shows the association of HCV status and several other baseline variables with risk of all-cause mortality among HIV infected veterans in a mutually adjusted multivariable regression model, with adjustment including baseline HIV RNA levels. Supplementary Table 2: shows the univariate association of several baseline variables with all-cause morality. Supplementary Table 3: shows the univariate association spresented under Supplementary Table 2, stratified by HCV status.

## Figures and Tables

**Table 1:** Baseline characteristics of HIV infected veterans included in analyses, by HCV status.

Variable	HCV+		HCV−		P value
	Number available	Mean (SD) or *N* (%)	Number available	Mean (SD) or *N* (%)	
Age (yrs)	5251	50 (7)	2788	49 (7)	<0.0001
Male	5251	5146 (98%)	2788	2745 (98%)	0.15
White	5251	1398 (27%)	2788	975 (35%)	<0.0001
Black	5251	3146 (60%)	2788	1510 (54%)	
Hispanic	5251	435 (8%)	2788	160 (6%)	
Smoking	5251	1068 (20%)	2788	480 (17%)	0.001
Hypertension	5251	1674 (32%)	2788	800 (29%)	0.003
Diabetes	5251	729 (14%)	2788	297 (11%)	<0.0001
CKD	5251	1100 (21%)	2788	520 (19%)	0.015
COPD	5251	404 (8%)	2788	167 (6%)	0.005
Anemia	5251	2718 (52%)	2788	1215 (44%)	<0.0001
CAD	5251	213 (4%)	2788	104 (4%)	0.48
PVD	5251	97 (2%)	2788	41 (1%)	0.22
Stroke	5251	68 (1% )	2788	34 (1%)	0.77
Cancer or history	5251	474 (9%)	2788	291 (10%)	0.04
DLD	4279	1093 (26%)	2347	465 (20%)	<0.0001
Drug abuse	5251	2052 (39%)	2788	513 (18%)	<0.0001
Alcohol abuse	5251	1451 (28%)	2788	434 (16%)	<0.0001
Major depression	5251	561 (11%)	2788	269 (10%)	0.15
BMI (kg/m^2^)	5143	24.7 (5.7)	2743	25.4 (4.5)	<0.0001
CD4 count (cells/uL)	3582	355 (290)	1972	366 (296)	0.15
HIV RNA (copies/uL)	2745	69812 (123540)	1379	70143 (128887)	0.94
Leukocyte count (cells/uL)	4973	5.5 (2.5)	2556	5.7 (2.5)	0.0005
AST (IU/L)	4996	70 (138)	2637	47 (115)	<0.0001
ALT (IU/L)	4979	64 (104)	2611	46 (120)	<0.0001
Albumin (g/dL)	4818	3.60 (0.68)	2447	3.87 (0.69)	<0.0001

ALT: alanine transaminase; AST: aspartate transaminase; BMI: body mass index; CAD: coronary artery disease; CKD: chronic kidney disease; COPD: chronic obstructive pulmonary disease; DLD: decompensated liver disease; PVD: peripheral vascular disease.

**Table 2:** Association of HCV status with mortality among HIV infected veterans in a multivariable regression model.

Variables	Model 1 *N* = 8030	Model 2 *N* = 4521	Full model *N* = 4521
HCV	1.78 (1.59, 1.96)	1.66 (1.44, 1.93)	1.58 (1.36, 1.84)
Age (per 10 yrs)	1.60 (1.51, 1.70)	1.51 (1.40, 1.64)	1.32 (1.21, 1.44)
Male	1.00 (0.70, 1.43)	1.07 (0.60, 1.89)	0.98 (0.55, 1.73)
DLD	—	3.73 (3.29, 4.24)	2.17 (1.88, 2.5)
Black versus white	—	—	0.86 (0.74, 1)
Hispanic versus white	—	—	0.79 (0.61, 1.03)
BMI (kg/m^2^)	—	—	0.94 (0.92, 0.95)
Smoking	—	—	0.97 (0.82, 1.14)
Hypertension	—	—	0.98 (0.85, 1.14)
Diabetes	—	—	1.45 (1.22, 1.71)
COPD	—	—	1.44 (1.19, 1.75)
Anemia	—	—	1.59 (1.36, 1.86)
CKD	—	—	1.74 (1.5, 2.02)
CAD	—	—	1.78 (1.42, 2.23)
Stroke	—	—	1.28 (0.87, 1.89)
PVD	—	—	0.82 (0.54, 1.23)
Thromboembolism	—	—	0.44 (0.11, 1.79)
Cancer	—	—	1.5 (1.26, 1.79)
Drug abuse	—	—	1.06 (0.9, 1.26)
Alcohol abuse	—	—	1.14 (0.96, 1.35)
Major depression	—	—	1.01 (0.82, 1.24)
CD4 count (per 100 clls/uL)	—	—	0.9 (0.88, 0.93)

Model 1: 8030 individuals with HIV infection had available data on HCV status, age, and sex.

Model 2: 4521 individuals with HIV infection had available data on HCV status, age, sex, and decompensated liver disease.

Model 3: 4521 individual with HIV infection had available data on all the 22 variables included in model.

ALT: alanine transaminase; AST: aspartate transaminase; BMI: body mass index; CAD: coronary artery disease; CKD: chronic kidney disease; COPD: chronic obstructive pulmonary disease; DLD: decompensated liver disease; PVD: peripheral vascular disease.

**Table 3:** Multivariate predictors of mortality in HIV infected individuals by HCV status.

Variables	HCV+ *N* = 2855	HCV− *N* = 1666
Age (per 10 yrs)	1.34 (1.21, 1.49)	1.2 (1, 1.45)
Male	1.02 (0.52, 1.98)	0.69 (0.22, 2.18)
Black versus white	0.72 (0.6, 0.87)	1.35 (1, 1.83)
Hispanic versus white	0.82 (0.62, 1.08)	0.38 (0.15, 0.95)
BMI	0.95 (0.94, 0.97)	0.89 (0.86, 0.92)
Smoking	0.91 (0.75, 1.1)	1.19 (0.84, 1.69)
Hypertension	0.96 (0.8, 1.14)	1.24 (0.92, 1.66)
Diabetes	1.25 (1.02, 1.52)	2.33 (1.67, 3.26)
DLD	2.33 (1.98, 2.74)	2.09 (1.57, 2.8)
COPD	1.57 (1.26, 1.95)	0.91 (0.58, 1.43)
Anemia	1.58 (1.31, 1.89)	1.49 (1.08, 2.07)
CKD	1.62 (1.36, 1.92)	1.99 (1.47, 2.71)
CAD	1.74 (1.32, 2.28)	1.81 (1.19, 2.73)
Stroke	0.99 (0.6, 1.64)	2.86 (1.48, 5.53)
PVD	0.98 (0.63, 1.53)	0.33 (0.12, 0.92)
Thromboembolism	0.79 (0.11, 5.75)	0.1 (0.01, 0.8)
Cancer	1.52 (1.23, 1.87)	1.55 (1.11, 2.18)
Drug abuse	1.2 (1, 1.43)	0.57 (0.36, 0.9)
Alcohol abuse	1.12 (0.93, 1.35)	1.05 (0.67, 1.65)
Major depression	1.04 (0.82, 1.31)	1.06 (0.66, 1.71)
CD4 count (per 100 clls/uL)	0.9 (0.87, 0.93)	0.92 (0.86, 0.97)
HCV treatment	0.41 (0.27, 0.63)	—

ALT: alanine transaminase; AST: aspartate transaminase; BMI: body mass index; CAD: coronary artery disease; CKD: chronic kidney disease; COPD: chronic obstructive pulmonary disease; DLD: decompensated liver disease; PVD: peripheral vascular disease.

**Table 4:** Association of HCV treatment with mortality by virologic response status in multivariate model.

Category	N	HR (95% CI)*
No HCV treatment	4,790	Ref.
Treated—no VR	116	0.40 (0.16–0.96)
Treated—VR	21	0.45 (0.06–3.20)
No HCV RNA data	332	0.41 (0.25–0.68)

VR: virologic response.

*HRs were similarly adjusted as in Table 3.
