# Supplementary material for: Predictors of Mortality among United States Veterans with Human Immunodeficiency Virus and Hepatitis C Virus Coinfection
Source: ISRN Gastroenterol. 2014 Apr 7;2014:764540. doi: 10.1155/2014/764540 (PMC4004106; doi:10.1155/2014/764540)
Supplement: Supplementary file 1 — Supplementary Tables: these materials provide supplementary information to the manuscript “Predictors of mortality among United States veterans with human immunodeficiency virus and hepatitis c virus coinfection”. Supplementary Table 1: shows the association of HCV status and several other baseline variables with risk of all-cause mortality among HIV infected veterans in a mutually adjusted multivariable regression model, with adjustment including baseline HIV RNA levels. Supplementary Table 2: shows the univariate association of several baseline variables with all-cause morality. Supplementary Table 3: shows the univariate association spresented under Supplementary Table 2, stratified by HCV status. [file 764540.f1.pdf]

Supplementary table 1: Association of HCV status with mortality among HIV infected Veterans in a multivariable regression model, including adjustment for baseline HIV RNA level.

| Variable | HR (95 % CI)<br>N = 2553 |
|---|---|
| HCV status | 1.47(1.21,1.79) |
| Age (per 10 yrs) | 1.3(1.16,1.47) |
| Male vs Female | 0.8(0.41,1.55) |
| Black vs White | 0.86(0.7,1.04) |
| Hispanic vs White | 0.88(0.63,1.24) |
| BMI | 0.94(0.92,0.96) |
| Smoking | 0.84(0.67,1.04) |
| Hypertension | 1.02(0.84,1.23) |
| Diabetes | 1.29(1.04,1.6) |
| DLD | 2.28(1.9,2.73) |
| COPD | 1.43(1.13,1.82) |
| Anemia | 1.43(1.16,1.75) |
| CKD | 1.86(1.53,2.25) |
| CAD | 2.32(1.77,3.03) |
| Stroke | 1.42(0.89,2.27) |
| PVD | 0.62(0.31,1.2) |
| Thromboembolism | 0.51(0.13,2.09) |
| Cancer | 1.5(1.2,1.89) |
| Drug abuse | 0.96(0.78,1.19) |
| Alcohol abuse | 1.24(1,1.55) |
| Major depression | 1.12(0.86,1.45) |
| CD4 count (per 100) | 0.93(0.89,0.96) |
| HIV RNA (per 1000) | 1.0009(1.0003,1.0014) |

Supplementary table 2: Association of several baseline variables with mortality in univariate Cox regression models

| Variable | HR (95% CI) | p-value |
|---|---|---|
| HCV positive | 1.76 (1.59,1.96) | <0.0001 |
| Age (per 10 yrs) | 1.63 (1.54-1.74) | <0.0001 |
| Male vs Female | 0.97 (0.68-1.39) | 0.18 |
| Black vs White | 1.18 (1.07, 1.31) | 0.001 |
| Hispanic vs White | 1.00 (0.84,1.22) | 0.93 |
| Body mass index | 0.92 (0.91, 0.93) | <0.0001 |
| Smoking | 1.15 (1.03, 1.29) | 0.01 |
| Hypertension | 1.31 (1.19,1.44) | <0.0001 |
| Diabetes | 1.59 (1.42,1.79) | <0.0001 |
| DLD | 3.80 (3.43,4.20) | <0.0001 |
| COPD | 1.93 (1.68,2.21) | <0.0001 |
| Anemia | 2.76 (2.51,3.05) | <0.0001 |
| CKD | 2.66 (2.42,2.92) | <0.0001 |
| CAD | 2.66 (2.26,3.13) | <0.0001 |
| Stroke | 2.15 (1.59,2.91) | <0.0001 |
| PVD | 1.61 (1.24,2.10) | 0.0004 |
| Venous thromboembolism | 0.90 (0.38,2.17) | 0.81 |
| Cancer | 1.78 (1.57,2.03) | <0.0001 |
| Drug abuse | 1.22 (1.12,1.34) | <0.0001 |
| Alcohol abuse | 1.23 (1.11,1.36) | <0.0001 |
| Major depression | 0.94 (0.82,1.09) | <0.0001 |
| CD4 count (per 100 cell/ul) | 0.85 (0.83,0.87) | <0.0001 |
| HCV treatment | 0.47 (0.36,0.60) | <0.0001 |

Supplementary table 3: Univariate predictors of mortality in HIV infected individuals by HCV status

| | HCV+ | P=value | HCV- | P-value |
|---|---|---|---|---|
| Age (per 10 yrs) | 1.60 (1.49-1.71) | <0.0001 | 1.60 (1.42-1.81) | <0.0001 |
| Male vs Female | 0.93 (0.63-1.36) | 0.18 | 1.55 (0.58-4.16) | 0.38 |
| Black vs White | 1.02 (0.91-1.15) | 0.91 | 1.49 (1.21-1.82) | <0.0001 |
| Hispanic vs White | 0.97 (0.79-1.12) | 0.74 | 0.61 (0.35-1.06) | 0.081 |
| BMI | 0.93(0.92,0.95) | 0.79 | 0.89(0.87,0.91) | 0 |
| Smoking | 1.09(0.96,1.23) | 0.20 | 1.25(0.99,1.58) | 0.066 |
| Hypertension | 1.25(1.12,1.39) | <0.0001 | 1.42(1.17,1.73) | <0.0001 |
| Diabetes | 1.47(1.29,1.67) | <0.0001 | 1.93(1.52,2.45) | <0.0001 |
| DLD | 3.54(3.15,3.98) | <0.0001 | 4.33(3.50,5.35) | <0.0001 |
| COPD | 1.90(1.63,2.21) | <0.0001 | 1.81(1.33,2.46) | <0.0001 |
| Anemia | 2.58(2.31,2.89) | <0.0001 | 3.08(2.52,3.77) | <0.0001 |
| CKD | 2.53(2.27,2.81) | <0.0001 | 3.12(2.56,3.80) | <0.0001 |
| CAD | 2.51(2.08,3.03) | <0.0001 | 3.22(2.34,4.44) | <0.0001 |
| Stroke | 1.63(1.11,2.38) | 0.012 | 4.02(2.44,6.62) | <0.0001 |
| PVD | 1.71(1.29,2.28) | <0.0001 | 1.19(0.61,2.30) | 0.61 |
| Thromboembolism | 1.87(0.60,5.81) | 0.28 | 0.74(0.18,2.98) | 0.67 |
| Cancer | 1.88(1.63,2.19) | <0.0001 | 1.79(1.40,2.30) | <0.0001 |
| Drug abuse | 1.12(1.01,1.24) | 0.029 | 0.95(0.74,1.22) | 0.68 |
| Alcohol abuse | 1.13(1.01,1.26) | 0.033 | 1.15(0.90,1.47) | 0.27 |
| Major depression | 0.96(0.81,1.12) | 0.59 | 0.79(0.56,1.12) | 0.18 |
| CD4 count (per 100) | 0.85(0.83,0.87) | <0.0001 | 0.83(0.79,0.87) | 0.000 |
| HCV treatment | 0.39(0.30,0.50) | <0.0001 | - | - |
